# Outcome and cognitive assessment of subjects over 65 years old admitted to the psychiatric ward

**DOI:** 10.1192/j.eurpsy.2025.1755

**Published:** 2025-08-26

**Authors:** F. Catalano, A. Di Lisi, M. Beghi, F. Cappella, M. Benassi, R. Raggini, R. P. Sant’Angelo

**Affiliations:** 1 Psychiatry, University of Bologna, Bologna; 2 Mental Health Department, Ausl Romagna; 3 Psychology, University of Bologna, Cesena, Italy

## Abstract

**Introduction:**

The psychogeriatric wards are managed by a specialized multidisciplinary team and are designed to provide a safe, comfortable, and therapeutically appropriate environment for patients with psychiatric issues, where, due to advanced age, pre-existing medical conditions, polypharmacy, cognitive impairments, and fragile physical conditions often coexist.

The elderly population, which is admitted in psychiatric ward in the absence of psychogeriatric wards, appears to be rather heterogeneous and in continuous grow. There are few studies in the literature that investigate the outcomes of hospitalization for these often fragile and vulnerable individuals in environments specific for the intensive management of acute psychopathological conditions.

**Objectives:**

This retrospective observational study aims to evaluate the effectiveness of clinical interventions in patients aged over 65 who are hospitalized in acute phases in the psychiatric ward at the Bufalini Hospital in Cesena.

**Methods:**

At the beginning and end of hospitalization, psychopathological symptoms were measured using the Brief Psychiatric Rating Scale (BPRS). Social and relational functioning was evaluated with the Health of the Nation Outcome Scales (HoNOS), while global cognitive performance was assessed through the Mini-Mental State Examination (MMSE) and the Clock Drawing Test (CDT).

**Results:**

The sample included 166 patients (91 F), with an average age of 71.2 years (SD 5.7). Among these, 128 (77.1%) completed tests. Patients were hospitalized for Bipolar Disorder (46), Psychotic symptoms (16), Major Depression (58), and Dementia (8).

From the general linear model for repeated measures, it emerged that the treatment administered during hospitalization led to a significant improvement in outcomes regardless of the admission diagnosis, for psychopathological symptoms, for socio-relational functioning, and for general cognitive functionality (MMSE and marginally p<0,05 CDT). There were no statistically significant differences related to diagnostic category, age, or gender.

**Conclusions:**

The present study provided important insights regarding the effectiveness of clinical interventions applied to patients over 65. The relevance of these results is particularly pronounced considering the increasing heterogeneity of the elderly population with psychiatric issues and the complexity of the treatments needed to address coexisting conditions and polypharmacy.

Further studies are needed to monitor clinical outcomes in the field of psychogeriatrics to further improve the quality of care, while also exploring the challenges related to the early differential diagnosis between psychiatric and dementia-related disorders, in order to ensure increasingly effective and personalized care.

**Disclosure of Interest:**

None Declared

